# LOESS-Based Cephalometric Growth Curves for Clinical Assessment of Craniofacial Development: A Cross-Sectional Study

**DOI:** 10.3390/dj14050269

**Published:** 2026-05-04

**Authors:** Luis Pablo Cruz-Hervert, Luis Cruz-Chávez, Jeta Kiseri-Kubati, Álvaro Edgar González-Aragón Pineda, Gerardo Martínez-Suárez, Carla Monserrat Ramírez-Martínez, Socorro Aída Borges-Yañez, Juan Carlos Solorio-Quezada, María Fernanda Trujillo-Sánchez, Silvia Paulina Martínez-Contreras, María-Eugenia Jiménez-Corona, Luis Fernando Jacinto-Alemán

**Affiliations:** 1 División de Estudios de Posgrado e Investigación, Facultad de Odontología, Universidad Nacional Autónoma de México, Coyoacan, Ciudad de México 04510, Mexico; aeoorto@gmail.com (L.P.C.-H.); dr.luis.cruz@gmail.com (L.C.-C.); c.ramirez@fo.odonto.unam.mx (C.M.R.-M.); aborges@unam.mx (S.A.B.-Y.); 2 Departamento de Epidemiología, Instituto Nacional de Cardiología Ignacio Chávez, Tlalpan, Ciudad de México 14080, Mexico; 3 Orthodontic and Dentofacial Department, University for Business and Technology, Pristina 10000, Kosovo; jkkubati@gmail.com; 4 Facultad de Estudios Superiores Iztacala, Universidad Nacional Autónoma de México, Tlalnepantla, México 54090, Mexico; alvaroedgar@unam.mx (Á.E.G.-A.P.); drsgema@yahoo.com.mx (G.M.-S.); 5 Escuela de Odontología, Universidad Anáhuac Campus Xalapa, Xalapa, Veracruz 91097, Mexico; dr_solorio@hotmail.com; 6 Departamento de Ortodoncia, Escuela Nacional de Estudios Superiores León, Universidad Nacional Autónoma de México, León, Guanajuato 37689, Mexico; mtrujillos@enes.unam.mx; 7 Especialidad de Ortodoncia, Universidad Cuauhtémoc, San Luis Potosí, San Luis Potosí 78290, Mexico; pau_mtz596@hotmail.com

**Keywords:** cephalometric, craniofacial growth, percentile curves, LOESS, growth trajectories

## Abstract

**Background/Objectives:** This cross-sectional study aimed to estimate the Locally Estimated Scatterplot Smoothing (LOESS)-smoothed percentiles for growth trajectories and evaluate age-related tendencies across groups using visual cross-sectional graphs. **Methods:** A total of 1147 patient records were analyzed, including 648 females and 469 males aged 5–20 years, with a mean age of 11.9 (SD ± 3.8) years. Twenty-seven cephalometric variables were organized into six measurement domains: cranial base, maxillary complex, mandibular complex, occlusal plane, vertical relationship, and sagittal relationship. Percentile curves were generated using LOESS regression across an age range of 5–20 years. **Results:** The LOESS-smoothed curves showed age-related trends across age groups. An upward trend in the curves was observed for the anterior and posterior cranial bases between 5 and 12 years of age, a plateau indicating reduced age-related change across groups during mid-adolescence. Maxillary measurements showed a similar pattern, with a clear upward tendency during childhood and reduced age-related change after approximately 12 years. Mandibular length and projection showed increasing trends during childhood, followed by a plateau or reduced slope across later age groups. The occlusal plane and vertical dimensions showed consistent patterns that approached a plateau around 12 years, indicating minimal age-related differences between groups. Changes in the ANB angle and Wits appraisal reflected a progressive forward tendency of the mandible across childhood age groups, followed by reduced age-related change during adolescence. **Conclusions:** These findings suggest that many craniofacial measurements show an upward trend during childhood followed by a plateau or reduced age-related change across age groups between approximately 12 and 14 years. The percentile-based growth curves presented here offer a practical reference for clinicians to evaluate craniofacial growth trajectories as population-level approximations derived from cross-sectional data in the pediatric population.

## 1. Introduction

Cephalometric norms support orthodontic diagnosis by enabling comparisons between individual craniofacial measurements and population references [1,2]. These norms help identify deviations from expected developmental patterns and guide treatment planning [3,4,5,6,7,8,9,10,11]. However, conventional cephalometric standards are typically expressed using means and standard deviations, which evaluate measurements individually and may not fully capture the integrated and proportional nature of craniofacial age-related variation across groups [1,2,7,12]. In addition, secular trends and cohort effects may influence craniofacial dimensions over time, suggesting that reference values may require periodic reassessment [13,14].

Craniofacial development is a dynamic and nonlinear biological process influenced by multiple factors during childhood and adolescence [1,2,4,6,12]. Several analytical approaches have been proposed to estimate expected values across age, including morphometric analyses, floating norms, and multivariate regression models [7,12,15,16,17,18]. While these methods have contributed to understanding craniofacial growth, their methodological complexity often limits their routine use in clinical settings.

Ideally, craniofacial growth patterns would be studied using longitudinal designs that follow individuals across developmental stages. However, in orthodontic research such studies are difficult to conduct without treatment-related confounding, and repeated radiographic monitoring of untreated individuals raises ethical concerns. Consequently, cross-sectional approaches are frequently used to describe population-level age-related patterns across groups.

Percentile-based approaches offer an alternative framework for evaluating growth patterns. Rather than relying solely on mean values, percentile curves describe the distribution of measurements across age and allow clinicians to interpret individual observations relative to the expected population range [19,20,21,22]. This approach facilitates the identification of upward trends, plateaus, or no clear age-related trend across groups in craniofacial measurements within clinical settings.

Locally Estimated Scatterplot Smoothing (LOESS) is a non-parametric regression technique that models age-related trends across observations without assuming a predefined functional form [16,23]. By applying localized smoothing across observed data points, LOESS can capture nonlinear patterns of variation across age groups commonly observed in craniofacial structures.

When applied to percentile estimation, LOESS allows the construction of smoothed growth curves representing population-level distributions across age ranges. Because these estimates are derived from cross-sectional observations rather than longitudinal follow-up, they should be interpreted as descriptive patterns across age groups that reflect trends in the data rather than direct observations of individual growth completion or maturation. Within this context, the resulting graphs provide a framework for contextualizing craniofacial measurements in similar clinical populations rather than serving as universal normative standards of craniofacial growth.

The aim of this cross-sectional study was to generate LOESS-smoothed percentile curves describing age-related patterns and growth trajectories inferred from cross-sectional data across a comprehensive set of cephalometric variables in children and adolescents from a Mexican clinical sample.

## 2. Materials and Methods

A secondary analysis was performed using a cross-sectional cephalometric design. Data were obtained from the clinical records of the Orthodontic Department, Postgraduate Studies and Research Division, Faculty of Dentistry, Universidad Nacional Autónoma de México. A random sample of pretreatment records was selected from patients who received orthodontic care between 2000 and 2016.

Records were included if patients had no prior orthodontic or orthopedic treatment, were non-syndromic, were between 4 and 20 years of age, and had lateral cephalometric radiographs of sufficient quality. The exclusion criteria comprised impacted, missing, or supernumerary teeth; a history of dental or skeletal trauma; and radiographic artifacts that could interfere with cephalometric assessment.

Following approval by the institutional ethics committee (CIE02/10/06/2016/04), 1147 lateral cephalograms were randomly selected from approximately 2500 pretreatment records. All radiographs were acquired using a PLANMECA (Planmeca, Helsinki, Finland) 2D unit (80–150 kHz, 50–84 kV) under a standardized imaging protocol routinely applied by the Department of Imaging. The patients were positioned in maximum intercuspation and instructed to remain still, with cephalic orientation standardized by aligning the Frankfort horizontal plane parallel to the floor and ensuring appropriate occlusal plane orientation.

Cephalometric measurements were obtained using WebCeph software Version: 1.0.5 (Assemble Circle Corporation, Seoul, Republic of Korea). All images were standardized using virtual calibration with a radiographic ruler. Landmark identification was initially performed automatically by the artificial intelligence algorithm integrated into the software. The resulting landmark positions were subsequently reviewed by a single experienced operator (L.C.C., >30 years of clinical experience) to confirm their accuracy. In a small number of cases where the automatic placement was clearly inaccurate, minor manual adjustments were made. These corrections were exceptional and performed only as part of quality control rather than as a systematic step in the landmarking process.

A total of 27 cephalometric variables were analyzed and grouped into six factors: cranial base, maxillary complex, mandibular complex, occlusal plane, vertical dimension, and sagittal relationship (Table 1). The reliability of WebCeph has been reported to be good to high in previous studies [15,16,17,24]. In the present analysis, the data quality was assessed by retracing 20 randomly selected radiographs at two separate time points. Intra- and inter-examiner reliability were evaluated using intraclass correlation coefficients (ICC) and the Dahlberg method, respectively. Most variables showed ICC values above 0.90, and the remaining exceeded 0.80, indicating excellent overall reliability.

### 2.1. Sample Size

As a non-parametric method, LOESS does not provide analytical variance estimates; therefore, bootstrap resampling (500 iterations) was applied to assess the stability of percentile estimates and to derive 95% confidence interval widths for the 25th percentile, 75th percentile, and IQR, following established recommendations for non-parametric smoothing and growth curve construction [23,25].

A target minimum of 32 observations per age group was adopted based on prior LOESS and bootstrap applications in growth modeling, indicating that approximately 30–40 observations per stratum yielded stable percentile estimates without excessive smoothing bias. When fewer observations were available, all data for that age group were retained. At this sample size, the percentile estimates remained stable across ages, while the increasing IQR values reflected the expected biological heterogeneity rather than sampling instability.

To facilitate the comparison of estimation precision across variables with different units and scales, a relative stability index was calculated as the ratio of the bootstrap-derived 95% confidence interval width of the IQR to the estimated IQR (wIQR/IQR), expressed as a percentage. Values between 80% and 100% indicated variability equal to or lower than expected for the given sample size, whereas values between 100% and 120% reflected acceptable uncertainty in the non-parametric percentile estimation. (Supplementary Figure S1)

Finally, sex-stratified LOESS growth curves could not be reliably estimated because stability analyses indicated that fewer than 32 observations per age group would produce biased percentile estimates. Consequently, the available sample size did not support sex-specific analyses with sufficient precision.

### 2.2. Statistical Methods

For the overall sample, the 10th, 25th, 50th, 75th, and 90th percentiles were estimated for each cephalometric variable by age. LOESS regression was used to construct percentile curves spanning 5 to 20 years, capturing age-related trends and facilitating visual assessment of developmental trajectories. Due to the wide range of values, percentile estimates are presented in Supplementary Table S1.

To support clinical interpretation, individual measurements were displayed using radar charts, enabling a simultaneous comparison of multiple parameters against reference percentiles. This visualization facilitates the assessment of individual growth trajectories, identification of deviations from expected patterns, and consideration of the need for further monitoring or intervention. All statistical analyses were performed using Stata version 17.0 (StataCorp, College Station, TX, USA).

### 2.3. Construction of LOESS-Based Growth Charts

Each LOESS-based growth chart was constructed using age as the independent variable and the corresponding cephalometric measurements as the dependent variable. Individual observations across ages 5–20 years were initially plotted to represent the raw cross-sectional distribution, after which LOESS regression was applied to model age-related trends without assuming linearity or a predefined functional form.

Percentile curves (10th, 25th, 50th, 75th, and 90th) were obtained by applying LOESS to age-specific percentile estimates, generating continuous percentile trajectories that defined growth channels across age. Locally adaptive smoothing allows the curves to capture both gradual changes and periods of stabilization in craniofacial development.

Each chart displays age on the horizontal axis and the measurement scale on the vertical axis, with spaces between adjacent percentile curves forming growth channels. Individual or group measurements, including repeated observations from the same subject, can be overlaid to visualize the growth direction, stability, or deviation relative to the expected age-related patterns.

This framework produces variable-specific growth charts that support population-level descriptions while enabling individual longitudinal assessments within a unified, non-parametric approach.

### 2.4. Ethical Approval and Consent to Participate

The study protocol was approved on 10 July 2016 by the Research and Ethics Committee of the Faculty of Dentistry of the National Autonomous University of Mexico (CIE02/10/06/2016/04). Anonymized data were obtained from a radiology image repository, with all personal identifiers removed to prevent linkage to individual participants. Images were handled exclusively by authorized personnel under a confidentiality agreement. As the dataset was fully anonymized and no identifiable information was included in the manuscript, the requirement for informed consent for publication was formally waived in accordance with the current ethical guidelines.

### 2.5. Use of Artificial Intelligence

Generative artificial intelligence tools were used solely to improve grammar, clarity, and overall writing quality. Specifically, ChatGPT (OpenAI, San Francisco, CA, USA) was employed for language refinement, and the online platform Rubriq (American Journal Experts, LLC, Winter Park, FL, USA) was used for the final evaluation of the writing quality and stylistic editing. No artificial intelligence tools were used for the data analysis, statistical modeling, or interpretation. The authors affirm that all data, ideas, analyses, interpretations, and conclusions presented in this manuscript are entirely their own.

## 3. Results

We analyzed 1147 patient records of individuals aged 5–20 years. Of these, 648 (58%) were female and 469 (42%) were male patients. The mean age was 11.9 ± 3.8 years old. Each age group included between 32 and 118 participants per group. To increase the sample size in early childhood, records from 4-year-old (n = 10) and 5-year-old (n = 25) patients were combined and analyzed as a single group. The distribution of participants by age and sex is shown in Table 2.

### 3.1. Clinical Utility of LOESS Growth Charts for Individual Growth Assessment

LOESS-based growth charts summarize age-related patterns for each cephalometric variable using percentile-defined growth channels. Each chart displays age on the horizontal axis and the corresponding measurement on the vertical axis, with channels defined by the 10th, 25th, 50th, 75th, and 90th percentiles to structure the distribution of values across age groups.

Individual observations can be plotted according to age and measurement value, and when repeated measurements are available, they can be displayed across successive ages within the same chart. This visualization allows individual data points to be interpreted relative to age-specific percentile channels for each variable.

Each chart represents a single cephalometric measurement, illustrating both its cross-sectional distribution and a smoothed age-related curve derived from cross-sectional observations. Collectively, these charts provide a graphical framework for describing population-level growth trajectories inferred from age-related trends across groups, while contextualizing individual observations within those patterns across the studied age ranges.

### 3.2. LOESS Growth Graphs for Cephalometric Measurements by Anatomical Factor

The results are presented for each cephalometric variable, with age-related trends across age groups described using LOESS-smoothed percentile curves. For clarity, the variables were organized and reported according to six predefined factors: cranial base, maxillary complex, mandibular complex, occlusal plane, vertical dimension, and sagittal relationship. This structure allows for a systematic description of age-related patterns and growth trajectories inferred from cross-sectional trends while preserving the clinical and anatomical coherence of the measurements.

#### 3.2.1. Factor 1: Cranial Base Features

##### Anterior Cranial Base (Sella–Nasion [SN])

**Figure 1:**
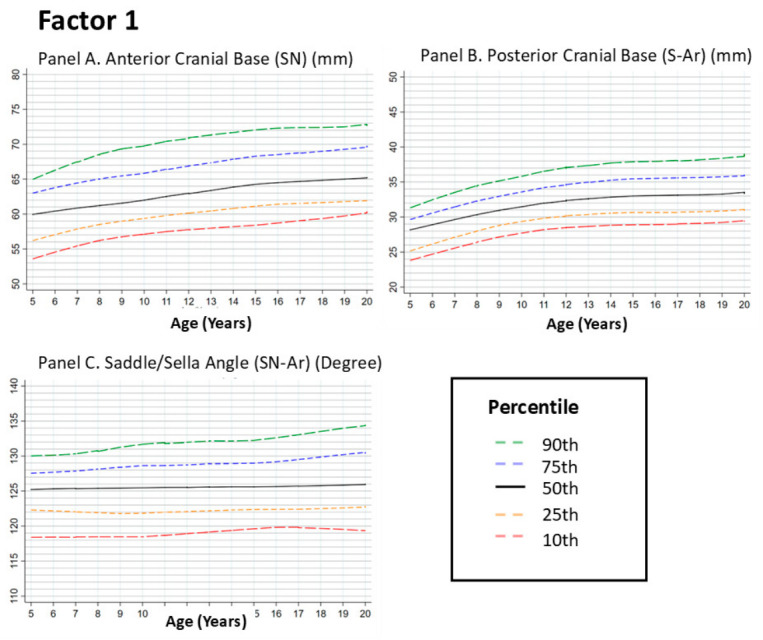
Growth Trajectories of the Posterior Cranial Base (S-Ar) and Saddle/Sella Angle (SN-Ar) from 5 to 20 Years. Footnote: Figure 1 illustrates the LOESS-smoothed percentile curves for anterior cranial base (Panel **A**), posterior cranial base (Panel **B**) and Saddle/Sella Angle (Panel **C**) in individuals aged 5–20 years. The curves show the 10th, 25th, 50th, 75th, and 90th percentiles of the data. The posterior cranial base showed an upward trend across age groups from early childhood to mid-adolescence, followed by a plateau with reduced age-related change after approximately 14 years. In contrast, the Saddle/Sella Angle demonstrated minimal changes over time, indicating a more stable craniofacial angular relationship throughout the growth period. These findings highlight the differential growth patterns of cranial base length and angular stability during development.

This panel illustrates age-related patterns in the length of the anterior cranial base (SN), a key component of craniofacial development. The LOESS curve showed an upward trend across age groups from 5 to approximately 12 years, corresponding to childhood and early adolescence. After this period, the curve approaches a plateau with reduced age-related change across groups (Figure 1, Panel A).

##### Posterior Cranial Base (Sella–Articulare [S–Ar])

The posterior cranial base showed a similar age-related pattern, with an upward trend during early age groups and a more evident increase toward early adolescence. During mid-adolescence (approximately 14–16 years), the curve shows a plateau indicating reduced differences across age groups. Clinically, this pattern suggests that posterior cranial base dimensions change less across later age groups, providing a structural context for mandibular development (Figure 1, Panel B).

##### Saddle (Sella) Angle (SN–Ar)

The saddle angle reflects the angular relationship between the anterior and posterior cranial bases. Across the examined age range, the percentile curves showed no clear age-related trend across groups, with only minor fluctuations. A plateau pattern appears early, generally before 12 years, and persists across later ages. This limited variation indicates relatively consistent angular relationships across the studied age groups (Figure 1, Panel C).

#### 3.2.2. Factor 2: Maxillary Features

##### Maxillary Depth (Frankfort Horizontal to Nasion–Point A)

**Figure 2:**
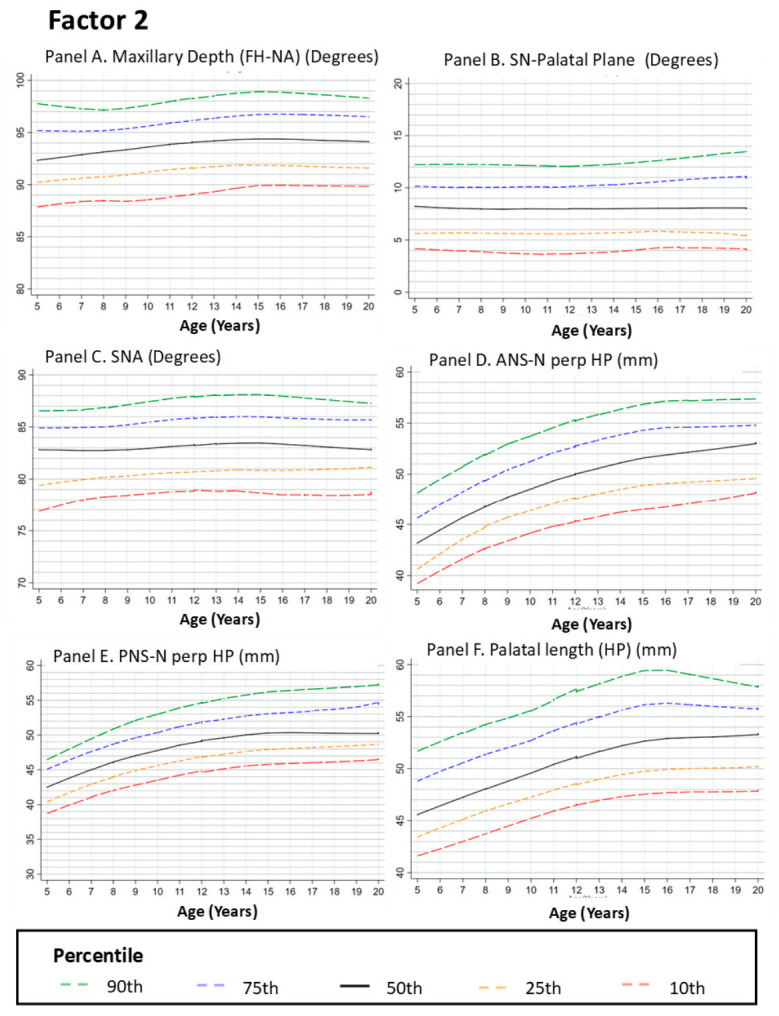
Growth Curves for Maxillary Measurements from Age 5 to 20. Footnote: Figure 2 presents LOESS-smoothed percentile curves for various maxillary dimensions, including maxillary depth (Panel **A**), SN-palatal plane (Panel **B**), SNA angle (Panel **C**), ANS_perp HP (Panel **D**), PNS-N perp HP (Panel **E**), and palatal length (Panel **F**). All panels display the 10th, 25th, 50th, 75th, and 90th percentiles. Panels **A**,**D**–**F** show upward trends across age groups for the linear measurements during childhood, followed by plateaus with reduced age-related change by mid-adolescence. In contrast, Panels **B**,**C** demonstrate more stable angular relationships throughout the growth period than those in Panel **A**. These findings emphasize the differential development of maxillary height and depth compared with angular stability, which contributes to overall craniofacial growth and alignment.

Maxillary depth relative to the cranial base showed an upward trend across age groups between 5 and 12 years, followed by reduced age-related change and a plateau after early adolescence (approximately 12–14 years). This pattern indicates smaller differences between later age groups in sagittal maxillary positioning (Figure 2, Panel A).

##### Sella–Nasion to Palatal Plane Angle (SN–PP)

The SN–PP angle reflects the maxillary inclination relative to the cranial base. The LOESS curves showed a gradual upward trend between 5 and 12 years, followed by a plateau with minimal age-related change across later age groups. This pattern suggests relatively consistent maxillary inclination across adolescence (Figure 2, Panel B).

##### SNA Angle

The SNA angle describes the sagittal relationship between the maxilla and anterior cranial base. The curves showed a modest upward trend from 5 to approximately 12 years, followed by a plateau indicating reduced age-related change across groups after early adolescence. This pattern reflects limited variation in maxillary–cranial base relationships across later age groups (Figure 2, Panel C).

##### Anterior Nasal Spine Perpendicular to the Horizontal Plane (ANS ⟂ HP)

This variable represents the vertical dimension of the anterior maxilla. The LOESS curves showed an upward trend during childhood and early adolescence, followed by a plateau between approximately 12 and 14 years, indicating reduced age-related change across later age groups (Figure 2, Panel D).

##### Posterior Nasal Spine Perpendicular to the Horizontal Plane (PNS ⟂ HP)

The vertical position of the posterior maxilla showed an upward trend during early age groups, reflecting larger differences in posterior maxillary height during childhood. After approximately 12 years, the curve shows a plateau with minimal variation across later age groups (Figure 2, Panel E).

##### Palatal Length (Posterior Nasal Spine [PNS]–Anterior Nasal Spine [ANS])

Palatal length showed an upward trend from 5 to approximately 14 years, with the most pronounced increase between 8 and 12 years. Beyond this age range, the curve approaches a plateau with minimal age-related change across groups (Figure 2, Panel F).

#### 3.2.3. Factor 3. Mandibular Features

##### Mandibular Length (Condylion–Gnathion [Co–Gn])

**Figure 3:**
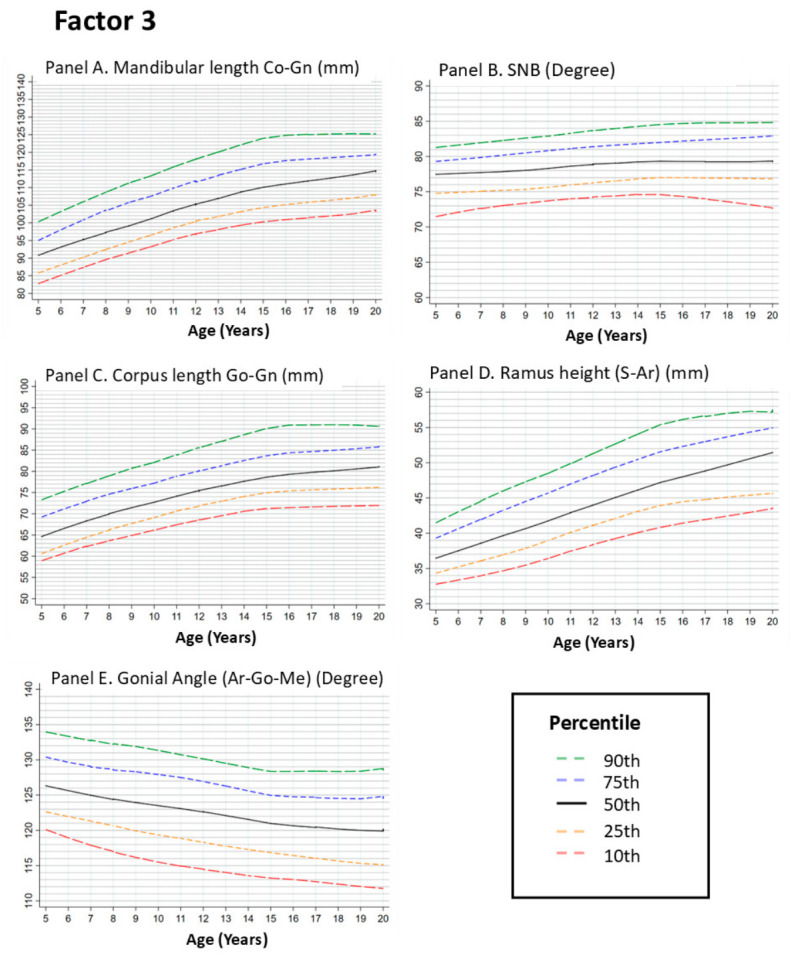
Growth Curves for Mandibular Measurements from Age 5 to 20. Footnote: Figure 3 illustrates the LOESS-smoothed percentile curves for mandibular dimensions, including mandibular length (Panel **A**), SNB angle (Panel **B**), corpus length (Panel **C**), ramus height (Panel **D**), and gonial angle (Panel **E**). All graphs show the 10th, 25th, 50th, 75th, and 90th percentiles. Panels **A**,**C**,**D** show upward trends in mandibular length and height across childhood age groups, followed by plateaus with reduced age-related change after approximately 14 years. Panel **B** shows a gradual increase in SNB, reflecting the mandibular projection. In contrast, Panel **E** highlights the relative stability of the gonial angle over time, with minor changes during adolescence. These findings underscore the differential growth rates of the mandibular body and angles, which contribute to overall facial harmony and occlusion.

Total mandibular length showed an upward trend across age groups between 5 and approximately 12 years, followed by a plateau with reduced age-related change during later age groups. This pattern indicates smaller differences in mandibular length across adolescence. Because mandibular length plays a key role in sagittal jaw relationships, variation in this measurement is relevant for interpreting maxillomandibular relationships (Figure 3, Panel A).

##### SNB Angle

The SNB angle reflects the sagittal relationship between the mandible and the cranial base and serves as an indicator of mandibular projection. The LOESS curves showed an upward trend from 5 to approximately 12–14 years, followed by a plateau indicating reduced age-related change across later age groups. This pattern suggests limited variation in mandibular projection across adolescence (Figure 3, Panel B).

##### Corpus Length (Gonion–Gnathion [Go–Gn])

Corpus length showed an upward trend between 5 and approximately 14 years, with the most pronounced increase between 8 and 12 years. After this period, the curve approaches a plateau with minimal age-related change across groups (Figure 3, Panel C).

##### Ramus Height (Sella–Articulare [S–Ar])

Ramus height showed an upward trend during childhood, with the curve increasing until approximately 12–14 years of age. Beyond this range, the curve shows a plateau with reduced age-related change across later age groups. This measurement contributes to mandibular vertical dimensions and overall facial proportions (Figure 3, Panel D).

In contrast to the linear mandibular measurements, the gonial angle showed no clear age-related trend across groups, with only minor fluctuations. The percentile curves remain relatively constant across the examined age range, indicating minimal variation in mandibular angular relationships across groups (Figure 3, Panel E).

#### 3.2.4. Factor 4. Occlusal Features

##### Mandibular Plane to Occlusal Plane (°)

**Figure 4:**
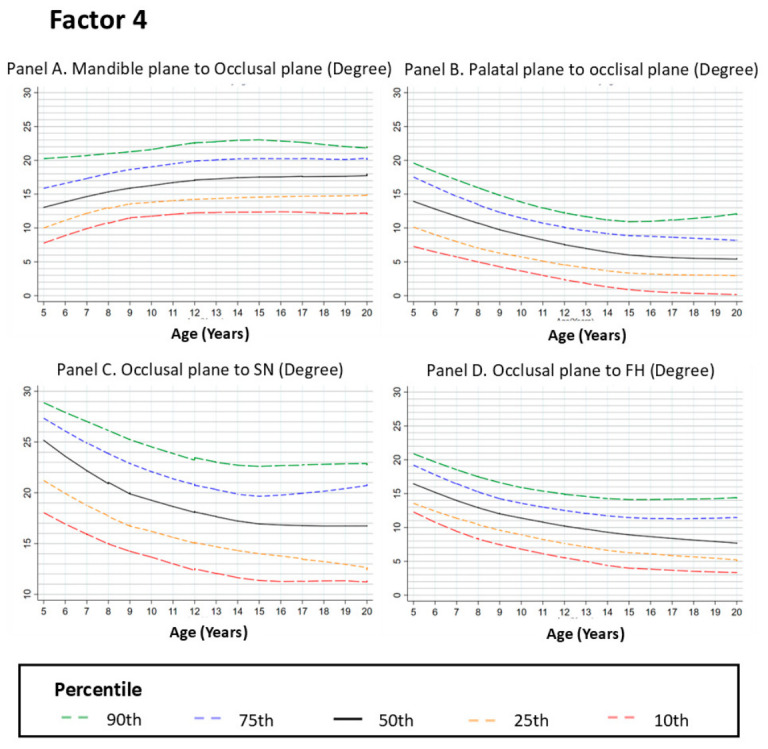
Growth Curves for Occlusal Plane Relationships from Ages 5 to 20. Footnote: Figure 4 shows the LOESS-smoothed percentile curves for occlusal plane relationships, including the mandible plane to occlusal plane (Panel **A**), palatal plane to occlusal plane (Panel **B**), occlusal plane to SN plane (Panel **C**), and occlusal plane to FH plane (Panel **D**). The curves represent the 10th, 25th, 50th, 75th, and 90th percentiles of the data. Panels **B**–**D** display a gradual decrease in angular relationships from ages 5 to 12, followed by plateaus with reduced age-related change across later age groups, suggesting an early adjustment of occlusal alignment. In contrast, Panel **A** indicates minor changes in the mandible to the occlusal plane relationship, stabilizing after age 12. These findings highlight the early development of occlusal and skeletal harmony during childhood and adolescence.

This angle describes the relationship between the mandibular and occlusal planes. The LOESS curves showed an upward trend across age groups between 5 and approximately 12 years, with the most evident change between 8 and 12 years. After this period, the curve approaches a plateau with reduced age-related change across later age groups (Figure 4, Panel A).

##### Palatal Plane to Occlusal Plane (°)

The angle between the palatal and occlusal planes showed a downward trend between 5 and approximately 12 years, followed by a mild upward trend across later age groups. Overall, the curves show reduced variation after early adolescence, indicating limited differences between later age groups (Figure 4, Panel B).

##### Occlusal Plane to Sella–Nasion Plane (°)

This parameter reflects the relationship between the occlusal plane and the cranial base. The curves showed a moderate downward trend between approximately 5 and 10 years, followed by reduced age-related change and a plateau across later age groups. This pattern indicates minimal variation in occlusal plane orientation relative to the cranial base during adolescence (Figure 4, Panel C).

##### Occlusal Plane to Frankfort Horizontal (FH) (°)

The angle between the occlusal plane and the Frankfort horizontal plane showed a downward trend between 5 and approximately 12 years, followed by a plateau with minimal age-related change across later age groups (Figure 4, Panel D).

#### 3.2.5. Factor 5. Vertical Dimension Features

##### Posterior Facial Height (Sella–Gonion [S–Go])

**Figure 5:**
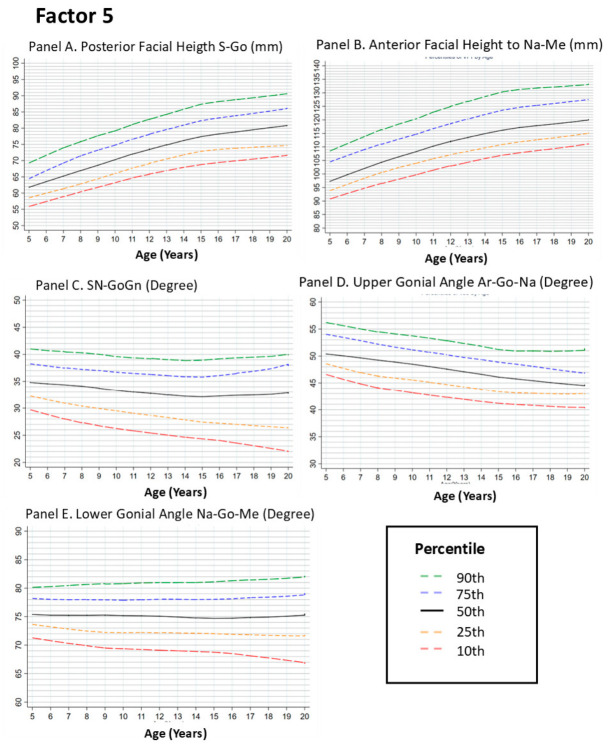
Growth Curves for Vertical Facial Measurements from Ages 5 to 20. Footnote: This figure presents LOESS-smoothed percentile curves for vertical facial dimensions, including posterior facial height (Panel **A**), anterior facial height (Panel **B**), SN-GoGn angle (Panel **C**), upper gonial angle (Panel **D**), and lower gonial angle (Panel **E**). The curves depict the 10th, 25th, 50th, 75th, and 90th percentiles of the data. Panels **A**,**B** show upward trends in facial height across age groups during childhood, followed by plateaus with minimal age-related change after approximately 14 years. In contrast, Panels **C**–**E** display relative stability in angular measurements, with minor variations throughout the growth period. These findings underscore the progressive nature of vertical facial growth and the early establishment of stable mandibular angles, which contribute to the overall facial balance and symmetry.

Posterior facial height reflects vertical dimensions of the posterior face. The LOESS curves showed an upward trend across age groups between 5 and approximately 12 years, followed by a plateau with reduced age-related change across later age groups. This pattern indicates smaller differences in posterior facial height across adolescence (Figure 5, Panel A).

##### Anterior Facial Height (Nasion–Menton [Na–Me])

Anterior facial height showed an upward trend between 5 and approximately 14 years, with the most evident increase between 8 and 12 years. After this period, the curve approaches a plateau with minimal age-related change across groups (Figure 5, Panel B).

##### SN–GoGn Angle

The SN–GoGn angle assesses mandibular plane inclination relative to the cranial base. The curves showed a gradual upward trend between 5 and approximately 12 years, followed by a plateau with reduced age-related change across later age groups. This pattern indicates limited variation in mandibular plane inclination during adolescence (Figure 5, Panel C).

##### Upper Gonial Angle (Articulare–Gonion–Nasion [Ar–Go–Na])

The upper gonial angle showed no clear age-related trend across groups, with only minor fluctuations between 5 and 10 years. The percentile curves remain relatively constant after early adolescence, indicating minimal variation in this angular relationship across age groups (Figure 5, Panel D).

##### Lower Gonial Angle (Nasion–Gonion–Menton [Na–Go–Me])

Similar to the upper gonial angle, the lower gonial angle showed no clear age-related trend across groups, with most variation occurring before approximately 12 years. After this age range, the curves show minimal change across later age groups (Figure 5, Panel E).

#### 3.2.6. Factor 6: SAGITTAL Relation Features

##### Convexity at Nasion–Pogonion (N–Pog)

**Figure 6:**
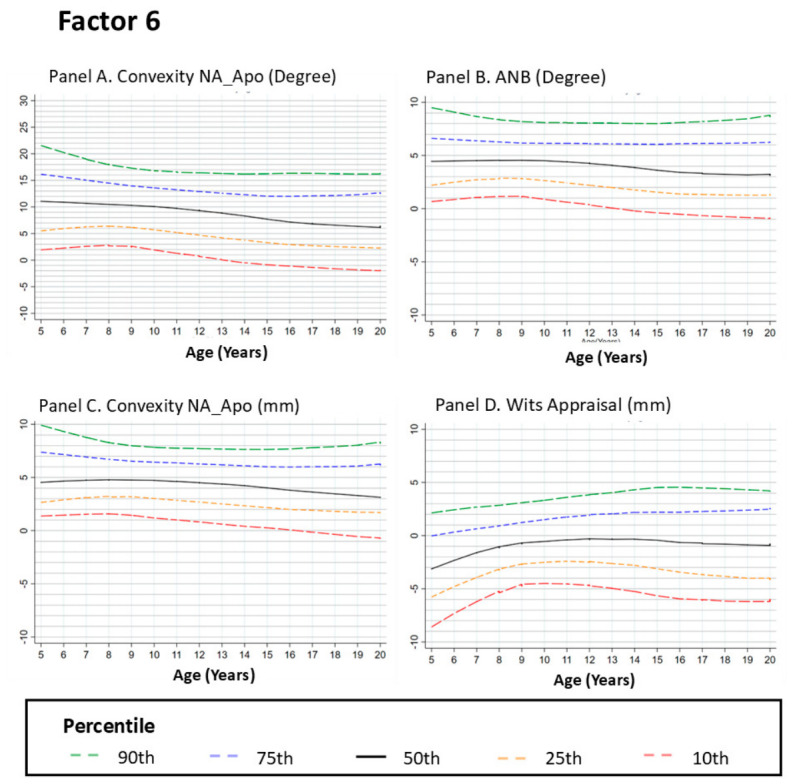
Growth Curves for Sagittal Convexity and Skeletal Relationships from Ages 5 to 20. Footnote: Figure 6 presents the LOESS-smoothed percentile curves for sagittal convexity and skeletal relationships, including convexity at NA-Apo (Panel **A**), ANB angle (Panel **B**), convexity at A-NPo (Panel **C**), and Wits appraisal (Panel **D**). The curves represent the 10th, 25th, 50th, 75th, and 90th percentiles of the data. Panels **A**,**C** show a gradual reduction in convexity from ages 5 to 12 years, with stabilization through adolescence, suggesting a relative forward tendency of the mandible across age groups. Panel **B** highlights the decrease in the ANB angle, reflecting improved alignment between the maxilla and mandible, whereas Panel **D** shows a downward trend in the Wits appraisal during childhood age groups followed by a plateau with minimal age-related change across later age groups.

Convexity along the nasion–pogonion line reflects the sagittal relationship between the lower face and cranial base. The LOESS curves showed a downward trend across age groups between 5 and approximately 12 years, followed by a plateau with reduced age-related change across later age groups. This pattern indicates smaller differences in lower facial convexity across adolescence (Figure 6, Panel A).

##### ANB Angle

The ANB angle evaluates the anteroposterior relationship between the maxilla and mandible relative to the nasion. The curves showed a slight downward trend between 5 and approximately 12 years, followed by a plateau with minimal age-related change across later age groups. This pattern indicates limited variation in sagittal maxillomandibular relationships across adolescence (Figure 6, Panel B).

##### Convexity at Point A

Convexity at point A reflects maxillary projection relative to the cranial base. The LOESS curves showed a modest downward trend between approximately 5 and 10 years, followed by a plateau with reduced age-related change across later age groups. This pattern indicates relatively consistent maxillary convexity across adolescence (Figure 6, Panel C).

##### Wits Appraisal

The Wits appraisal quantifies sagittal jaw relationships by measuring the linear distance between points A and B projected onto the occlusal plane. The curves showed a downward trend across age groups during childhood until approximately 12 years, followed by a plateau indicating minimal age-related change across later age groups (Figure 6, Panel D).

## 4. Discussion

The principal contribution of our study lies in the development of percentile-based growth curves derived from cross-sectional observations, offering clinicians a practical framework for evaluating age-related craniofacial trends across age groups in pediatric and adolescent orthodontic patients. By moving beyond the traditional reliance on mean values and standard deviations, this approach incorporates percentile-based visualization of craniofacial measurements, making interpretation more intuitive and clinically applicable. Because the dataset was derived from pretreatment orthodontic records, the resulting curves should be interpreted as clinic-based reference patterns rather than universal normative standards of craniofacial growth. Accordingly, these curves are best used for contextualizing craniofacial measurements within similar clinical populations and settings. Within this context, the study provides a useful framework for understanding population-level growth trajectories inferred from age-related trends across groups.

### 4.1. Cranial Base Growth

Our percentile curves show an upward trend across age groups between approximately 5 and 12 years for both the anterior and posterior cranial bases, followed by a plateau with reduced age-related change during mid-adolescence. At the population level, this pattern suggests that cranial base measurements show smaller differences across later age groups, providing a structural context for subsequent maxillary and mandibular development [26,27].

### 4.2. Maxillary Growth

Maxillary dimensions, assessed using parameters such as the SNA angle and palatal length, showed an upward trend across age groups during childhood, followed by reduced age-related change after approximately 12 years. This pattern is consistent with previous reports showing limited variation in SNA values across later age groups [28]. Within a cross-sectional context, these trends may approximate the timing at which changes in maxillary measurements become less pronounced across age groups, which has implications for the timing of orthodontic interventions.

### 4.3. Mandibular Growth

Mandibular length (Go–Gn) and projection (SNB angle) showed an upward trend across age groups during childhood, followed by a plateau with reduced age-related change after approximately 12 years. Compared with maxillary measurements, the curves suggest that mandibular variables may show greater variation across later age groups. This difference highlights the importance of continued monitoring of mandibular measurements throughout adolescence. Comparable patterns have been described in previous studies reporting continued variation in SNB and mandibular length across adolescence [29].

### 4.4. Occlusal and Vertical Relationships

Analysis of occlusal plane parameters and vertical dimensions, including anterior and posterior facial heights, showed upward trends during childhood followed by plateaus across later age groups. These patterns suggest reduced age-related variation in these measurements after early adolescence. Previous studies have reported similar reductions in age-related change for vertical facial measurements when compared with sagittal variables [29].

### 4.5. Sagittal Relationships

Assessment of sagittal relationships using the ANB angle and Wits appraisal showed downward trends across age groups during childhood, followed by plateaus with minimal age-related change after approximately 12 years. At the population level, these patterns indicate reduced variation in sagittal jaw relationships across later age groups. Similar age-related reductions in ANB values across adolescence have been reported previously [28].

### 4.6. Limitations

The following limitations should be interpreted as inherent to the study design and methodological approach. The cross-sectional design allows for the characterization of population-level growth patterns but does not capture individual longitudinal trajectories. As a result, conclusions regarding the timing and apparent stabilization of craniofacial growth between 12 and 14 years of age should be interpreted as reflecting population trends rather than precise individual developmental milestones. Longitudinal designs are more appropriate for describing individual growth dynamics and transitional phases over time.

The use of LOESS-smoothed percentile curves provides a flexible and visually intuitive representation of age-related trends, supporting exploratory analyses and the construction of clinically meaningful growth channels [30]. Nevertheless, because LOESS is a non-parametric smoothing technique, it does not model the full underlying distribution of craniofacial variables in the same way as the Lambda Mu Sigma Method (LMS) or Generalized Additive Models for Location, Scale, and Shape (GAMLSS) approaches, which limits the precision of percentile estimation [31,32]. Accordingly, the curves generated in this study should be interpreted as descriptive and clinically oriented tools rather than definitive normative references, particularly when inferring the exact thresholds of skeletal maturation.

Another methodological consideration with direct clinical relevance is the absence of sex-stratified analyses. Although the overall sample size was sufficient to generate robust population-level percentiles, it did not allow for fully reliable sex-specific modeling without increasing the risk of bias. Consequently, potential differences in the timing or magnitude of craniofacial growth between males and females were not formally examined, which may limit the application of the proposed curves in contexts in which sex-specific growth patterns are clinically relevant.

The characteristics of the study sample reflect the methodological framework of this investigation. The population consisted of pretreatment orthodontic patients from a single geographical region (2000–2016), which may introduce referral bias and limit external validity. Because this clinic-based sample may over-represent individuals with malocclusion or craniofacial disproportions, the distribution of some measurements may differ from that of the general population and may influence the position of the estimated percentiles. Therefore, the curves presented here should be interpreted as clinical reference patterns derived from an orthodontic population rather than normative standards for the general population. Additionally, the ethnic and geographical homogeneity of the sample further limits generalizability. Future research should include longitudinal designs, more diverse populations, and sex-specific percentile curves to better characterize craniofacial growth patterns.

## 5. Conclusions

This study presents a novel set of LOESS-smoothed percentile curves for key craniofacial parameters, providing clinicians with an intuitive and clinically accessible framework for assessing growth adequacy in children and adolescents. By describing age-related tendencies through percentile-based growth channels, these curves allow for the monitoring of whether individual trajectories follow the expected phases of increase, stabilization, or deviation from typical patterns.

The results indicated that most craniofacial structures exhibit marked upward growth during childhood, followed by a stabilization phase that typically occurs between 12 and 14 years of age. This finding underscores the clinical relevance of early growth assessment and timely intervention in cases of malocclusion or disproportionate development, as most dynamic changes occur before adolescence.

Future research should prioritize longitudinal study designs and the inclusion of ethnically and geographically diverse populations to validate individual growth trajectories beyond cross-sectional approximations. The broader contribution of this study lies in bridging population-based growth assessment with clinically practical tools, supporting more personalized, timely, and effective decision-making in orthodontic and craniofacial growth management. Accordingly, the percentile curves presented here are best used to contextualize craniofacial measurements within similar populations and clinical settings rather than as universal normative standards.

## Figures and Tables

**Table 1:** Cephalometric variable definitions by craniofacial factor.

Factor	Variable	Definition
1. Cranial base	Sella–Nasion (SN)	Linear distance from S to N (mm)
	S–Articulare (S–Ar)	Linear distance from S to Ar (mm)
	Saddle/Sella Angle (SN–Ar)	Angle between SN and Ar lines (°)
2. Maxillary complex	SNA	Angle between Sella–Nasion line and Point A (°)
	ANS-N to HP	Perpendicular distance from ANS to horizontal plane (mm)
	PNS–ANS	Palatal length (mm)
	SN–PP	Angle between SN and palatal plane (°)
	PNS–N to HP	Distance from PNS to horizontal plane (mm)
	A–FH	Linear distance from Point A to Frankfort plane (mm)
3. Mandibular complex	Go–Gn	Linear distance from Gonion to Gnathion (mm)
	Co–Gn	Linear distance from Condylion to Gnathion (mm)
	Ar–Go	Linear distance from Articulare to Gonion (mm)
	Ar–Go–Me	Gonial angle (°)
	SNB	Angle between SN and Point B (°)
4. Occlusal plane	MP–OP	Angle between mandibular and occlusal planes (°)
	PP–OP	Angle between palatal and occlusal planes (°)
	OP–SN	Angle between occlusal and SN planes (°)
	OP–FH	Angle between occlusal and FH planes (°)
5. Vertical dimension	S–Go	Posterior facial height (mm)
	Na–Me	Anterior facial height (mm)
	SN–GoGn	Angle between SN and mandibular plane (°)
	Ar–Go–Na	Upper gonial angle (°)
	Na–Go–Me	Lower gonial angle (°)
6. Sagittal relationships	ANB	Angle between points A, N, and B (°)
	Wits appraisal	Distance between A and B points projected on occlusal plane (mm)
	Convexity at Point A	Angle formed by N–A–Pg (°)
	Convexity (N–Pog)	Linear measurement from Nasion to Pogonion (mm)

**Table 2:** Sample Distribution by Age Group and Sex.

Age (Years)	Female		Male		Total	
	Frequency	%	Frequency	%	Frequency	%
5	18	51.4%	17	48.6%	35	3.1%
6	23	54.8%	19	45.2%	42	3.7%
7	36	56.3%	28	43.8%	64	5.6%
8	47	53.4%	41	46.6%	88	7.7%
9	52	54.2%	44	45.8%	96	8.4%
10	69	62.2%	42	37.8%	111	9.7%
11	70	59.3%	48	40.7%	118	10.3%
12	73	64.6%	40	35.4%	113	9.9%
13	68	59.6%	46	40.4%	114	9.9%
14	48	51.6%	45	48.4%	93	8.1%
15	35	63.6%	20	36.4%	55	4.8%
16	33	66.0%	17	34.0%	50	4.4%
17	21	44.7%	26	55.3%	47	4.1%
18	28	66.7%	14	33.3%	42	3.7%
19	16	50.0%	16	50.0%	32	2.8%
20	30	63.8%	17	36.2%	47	4.1%
Total	667	58.2%	480	41.8%	1147	100.0%

## Data Availability

The dataset is available on a well-founded and adequate request from the authors.

## Supplementary Materials

The following supporting information can be downloaded at: https://www.mdpi.com/article/10.3390/dj14050269/s1. Table S1: Estimated values for sample size calculation. Figure S1: Sample size adequacy assessment using bootstrap-derived interquartile range stability.

## Abbreviations

The following abbreviations are used in this manuscript:

AI	Artificial intelligence
ANS	Anterior nasal spine
ANB	Angle formed by points A, N, and B
Ar	Articulare
Co	Condylion
FH	Frankfort horizontal plane
Go	Gonion
Gn	Gnathion
HP	Horizontal plane
ICC	Intraclass correlation coefficient
IQR	Interquartile range
LOESS	Locally Estimated Scatterplot Smoothing
Me	Menton
Na	Nasion
OP	Occlusal plane
Pg/Pog	Pogonion
PNS	Posterior nasal spine
PP	Palatal plane
S	Sella
SD	Standard deviation
SN	Sella–Nasion
SNA	Angle between Sella–Nasion line and point A
SNB	Angle between Sella–Nasion line and point B
SN–GoGn	Angle between Sella–Nasion and mandibular plane
Wits	Wits appraisal
