# Supplementary material for: LOESS-Based Cephalometric Growth Curves for Clinical Assessment of Craniofacial Development: A Cross-Sectional Study
Source: Dent J (Basel). 2026 May 4;14(5):269. doi: 10.3390/dj14050269 (PMC13205012; doi:10.3390/dj14050269)
Supplement: Supplementary file 1 [file dentistry-14-00269-s001.zip › Suplementary table S1 v2.pdf]

Suplementary table S1. Estimated values for sample size calculation

Factor	Cephalomatric variable	Age (Years)	n	Estimated 25th	Estimated 75	Estimated IQR	Width of 25th	Width of P75	Width of QR	Width QR/ Estimated IQR
Factor 1	Saddle/Sella Angle	5	32	121.6	127	5.4	5.6	5.2	7	129%
Factor 1	Saddle/Sella Angle	6	32	122.3	128	5.7	5.4	2.8	5.4	94%
Factor 1	Saddle/Sella Angle	7	32	122.5	128.5	5.9	4.3	2.4	4.2	71%
Factor 1	Saddle/Sella Angle	8	32	121.2	127.8	6.6	6	2	5.8	88%
Factor 1	Saddle/Sella Angle	9	32	121.8	127.4	5.6	4.6	4.8	5.9	105%
Factor 1	Saddle/Sella Angle	10	32	121.6	129.9	8.3	5.4	5.5	6.2	75%
Factor 1	Saddle/Sella Angle	11	32	121.9	128.7	6.8	5.2	5.6	6	89%
Factor 1	Saddle/Sella Angle	12	32	121.4	129.3	7.9	5.3	5.2	6.6	83%
Factor 1	Saddle/Sella Angle	13	32	123.2	129.1	5.9	5	4.1	5.4	91%
Factor 1	Saddle/Sella Angle	14	32	122.5	127.5	4.9	3.7	4.6	4.7	96%
Factor 1	Saddle/Sella Angle	15	32	122.3	128	5.7	3.2	4.3	4.3	75%
Factor 1	Saddle/Sella Angle	16	32	121.9	130.6	8.8	5.3	4	5.4	61%
Factor 1	Saddle/Sella Angle	17	32	122.6	130	7.4	4.5	5.7	6.2	84%
Factor 1	Saddle/Sella Angle	18	32	121.5	129.1	7.7	4.7	5.5	6.4	84%
Factor 1	Saddle/Sella Angle	19	32	123.1	130.3	7.2	4.5	6.9	6.7	94%
Factor 1	Saddle/Sella Angle	20	32	122.9	130.5	7.6	4.3	5.2	5.5	73%
Factor 1	Anterior Cranial Base	5	32	56.2	62.7	6.5	5.1	2.8	5.7	87%
Factor 1	Anterior Cranial Base	6	32	56.3	63	6.6	3.8	4.3	5.1	76%
Factor 1	Anterior Cranial Base	7	32	58.3	65.3	7	3.5	6.8	6.4	92%
Factor 1	Anterior Cranial Base	8	32	58.7	66	7.3	4.3	5.4	5.8	79%
Factor 1	Anterior Cranial Base	9	32	59.6	64.9	5.3	3.4	5.4	5.3	100%
Factor 1	Anterior Cranial Base	10	32	58.8	65.5	6.7	3.1	4.8	4.8	72%
Factor 1	Anterior Cranial Base	11	32	59.4	66.4	7.1	2.9	7	6.5	92%
Factor 1	Anterior Cranial Base	12	32	60.5	66.2	5.7	3.9	3.8	4.7	82%
Factor 1	Anterior Cranial Base	13	32	60.5	68	7.6	3.9	6.1	5.7	75%
Factor 1	Anterior Cranial Base	14	32	61.4	69.6	8.2	4.6	7.2	6.5	80%
Factor 1	Anterior Cranial Base	15	32	60.4	68.5	8.1	5.3	5.4	6.5	80%
Factor 1	Anterior Cranial Base	16	32	60.6	67.3	6.7	3.3	5.8	5.8	86%
Factor 1	Anterior Cranial Base	17	32	61.6	69.3	7.7	6.6	4.8	6.5	84%
Factor 1	Anterior Cranial Base	18	32	62.2	69	6.8	3	8	7.2	105%
Factor 1	Anterior Cranial Base	19	32	63.8	68.5	4.7	3.2	6	6.2	132%
Factor 1	Anterior Cranial Base	20	32	60.6	70.5	9.9	3.9	5.6	6.6	67%
Factor 1	Posterior Cranial Base	5	32	25.5	29.2	3.7	3.9	1.7	3.7	100%

Factor 1	Posterior Cranial Base	6	32	25.7	30.6	4.9	3.5	3.2	4.7	96%
Factor 1	Posterior Cranial Base	7	32	26.9	32.1	5.2	3.2	2.8	3.8	75%
Factor 1	Posterior Cranial Base	8	32	28.4	32.7	4.2	2.8	3.3	3.5	83%
Factor 1	Posterior Cranial Base	9	32	29	32.6	3.6	3	3.1	3.9	107%
Factor 1	Posterior Cranial Base	10	32	29.4	34	4.6	2.1	3.4	3	66%
Factor 1	Posterior Cranial Base	11	32	30.2	33.7	3.6	2.2	2.6	2.7	75%
Factor 1	Posterior Cranial Base	12	32	29.7	35	5.3	2.6	3.5	3.7	70%
Factor 1	Posterior Cranial Base	13	32	31.1	35.5	4.4	3.1	3.9	3.9	88%
Factor 1	Posterior Cranial Base	14	32	31.4	36.3	4.9	3.3	3.9	4	81%
Factor 1	Posterior Cranial Base	15	32	29.1	35.5	6.4	4.2	4.1	5.1	80%
Factor 1	Posterior Cranial Base	16	32	30.6	34	3.4	2.3	2.5	2.9	85%
Factor 1	Posterior Cranial Base	17	32	31	35.8	4.8	3.1	3.9	4.2	88%
Factor 1	Posterior Cranial Base	18	32	30.8	36.3	5.5	2.9	3.6	3.9	71%
Factor 1	Posterior Cranial Base	19	32	30.2	35.3	5.1	3	4.6	4.7	93%
Factor 1	Posterior Cranial Base	20	32	31.3	36.1	4.8	2.6	3.2	3.5	73%
Factor 2	Maxillary Depth	5	32	89.7	95.2	5.5	1.9	4.3	5.2	94%
Factor 2	Maxillary Depth	6	32	90.7	95.4	4.8	3.6	1.7	3.6	76%
Factor 2	Maxillary Depth	7	32	90.6	95	4.4	3.8	3	4.5	102%
Factor 2	Maxillary Depth	8	32	90.8	95.1	4.3	2.6	3	3.4	79%
Factor 2	Maxillary Depth	9	32	90.9	95	4.1	3.5	2.6	3.7	92%
Factor 2	Maxillary Depth	10	32	90.2	95.8	5.6	4.7	2.5	4.5	81%
Factor 2	Maxillary Depth	11	32	92.1	96	4	3.6	2.6	3.9	100%
Factor 2	Maxillary Depth	12	32	92	96	4	3.2	2.4	3.4	86%
Factor 2	Maxillary Depth	13	32	91.9	96.6	4.7	3.7	4	4.6	97%
Factor 2	Maxillary Depth	14	32	92.1	96.7	4.6	2.5	3.3	3.4	74%
Factor 2	Maxillary Depth	15	32	92	97.5	5.5	3.3	3.8	4.1	75%
Factor 2	Maxillary Depth	16	32	91.9	96.4	4.6	3.7	4	4.4	96%
Factor 2	Maxillary Depth	17	32	90.7	96.8	6.1	3.3	4	4.7	78%
Factor 2	Maxillary Depth	18	32	92.5	97.1	4.6	2	3.1	2.9	63%
Factor 2	Maxillary Depth	19	32	91.8	96.3	4.5	5.5	3	4.9	109%
Factor 2	Maxillary Depth	20	32	91.4	96.5	5.1	2.7	2.2	3.3	65%
Factor 2	SN-Palatal Plane	5	32	5.6	10.3	4.7	3	3.2	3.3	71%
Factor 2	SN-Palatal Plane	6	32	5.8	10.2	4.4	3	2	3	66%
Factor 2	SN-Palatal Plane	7	32	5.2	10.2	4.9	2.7	3.3	3.5	72%
Factor 2	SN-Palatal Plane	8	32	6	9.4	3.5	2.9	3.8	4	115%
Factor 2	SN-Palatal Plane	9	32	5.7	10.5	4.8	3	2.8	3.4	72%
Factor 2	SN-Palatal Plane	10	32	5.3	10.2	4.9	3.3	3.6	4.2	86%
Factor 2	SN-Palatal Plane	11	32	5.5	10	4.5	3.3	2.9	3.7	82%
Factor 2	SN-Palatal Plane	12	32	5.9	10.6	4.8	3.6	2.8	3.5	73%
Factor 2	SN-Palatal Plane	13	32	5.3	10.1	4.8	3.6	3.3	4	83%
Factor 2	SN-Palatal Plane	14	32	5.5	8.9	3.4	2.8	2.9	3.3	96%

Factor 2	SN-Palatal Plane	15	32	5.6	11	5.4	2.2	3.4	3.5	65%
Factor 2	SN-Palatal Plane	16	32	6.1	10.8	4.8	3.1	3.6	4.1	86%
Factor 2	SN-Palatal Plane	17	32	5.9	12.1	6.2	3.3	4	4.7	76%
Factor 2	SN-Palatal Plane	18	32	6.2	10.2	4	2.3	3	3	76%
Factor 2	SN-Palatal Plane	19	32	6.4	11.1	4.7	2.7	4.2	3.9	84%
Factor 2	SN-Palatal Plane	20	32	4.7	10.9	6.2	4.1	4.6	5.9	95%
Factor 2	ANS-Perp HP	5	32	39.6	45.1	5.5	3	3.2	3.6	65%
Factor 2	ANS-Perp HP	6	32	42.7	46.7	4	2.6	3.2	3.5	88%
Factor 2	ANS-Perp HP	7	32	43.6	48.8	5.2	3.3	5.4	4.8	91%
Factor 2	ANS-Perp HP	8	32	45.5	50.1	4.6	1.9	3.8	3.8	82%
Factor 2	ANS-Perp HP	9	32	45.9	49.7	3.8	3.2	3	3.3	86%
Factor 2	ANS-Perp HP	10	32	46.6	51.7	5.1	3.4	4.1	4.9	96%
Factor 2	ANS-Perp HP	11	32	46.5	52.3	5.8	2.9	3.4	3.1	53%
Factor 2	ANS-Perp HP	12	32	47.7	52.2	4.5	2.4	3.8	3.6	80%
Factor 2	ANS-Perp HP	13	32	48.5	53.9	5.4	3.2	3.9	4.3	79%
Factor 2	ANS-Perp HP	14	32	49.3	55.5	6.2	3.9	3.9	4.6	74%
Factor 2	ANS-Perp HP	15	32	49.4	53.5	4.1	3.6	4.7	5	123%
Factor 2	ANS-Perp HP	16	32	47.8	53.2	5.4	4.4	2.5	4.1	75%
Factor 2	ANS-Perp HP	17	32	50	55.4	5.4	4.9	4.3	5.3	98%
Factor 2	ANS-Perp HP	18	32	48.7	55.7	7.1	2.7	4.7	4.7	67%
Factor 2	ANS-Perp HP	19	32	50	55	5	3.8	3.2	4.3	85%
Factor 2	ANS-Perp HP	20	32	49.4	54.2	4.8	3.3	2.3	3.5	74%
Factor 2	PNS-N perp HP	5	32	39.5	44	4.5	3.2	4	4.6	103%
Factor 2	PNS-N perp HP	6	32	42	46.8	4.8	2.2	2.9	3.1	64%
Factor 2	PNS-N perp HP	7	32	43.3	48	4.7	3.3	2.3	3.4	73%
Factor 2	PNS-N perp HP	8	32	44.7	49.8	5.1	2.7	4	3.9	78%
Factor 2	PNS-N perp HP	9	32	44.6	48.7	4.1	2.9	3.4	4	98%
Factor 2	PNS-N perp HP	10	32	45.6	50.6	5	3.3	5	4.9	98%
Factor 2	PNS-N perp HP	11	32	46.2	51.2	5	3.6	4	4.3	87%
Factor 2	PNS-N perp HP	12	32	47.2	51.4	4.3	3	4.6	4.7	111%
Factor 2	PNS-N perp HP	13	32	47.4	53.5	6.1	3.3	3.9	4.4	71%
Factor 2	PNS-N perp HP	14	32	49	54.6	5.6	3.5	4.9	4.6	82%
Factor 2	PNS-N perp HP	15	32	47	52.8	5.8	4	4.3	5.3	91%
Factor 2	PNS-N perp HP	16	32	47.9	51.9	4	2.8	3.8	4.4	112%
Factor 2	PNS-N perp HP	17	32	48.2	52.7	4.5	2.3	6.1	5.3	118%
Factor 2	PNS-N perp HP	18	32	48.1	53.9	5.8	2.5	5.4	5.3	91%
Factor 2	PNS-N perp HP	19	32	48.8	54.5	5.7	2.7	3.8	4	71%
Factor 2	PNS-N perp HP	20	32	48.7	54.8	6.1	3.3	6	5.9	97%
Factor 2	Palatal Length	5	32	42.7	47.7	5	2.3	3.1	2.8	57%
Factor 2	Palatal Length	6	32	44.3	50	5.7	3	6.6	6.4	113%
Factor 2	Palatal Length	7	32	45.7	51.2	5.5	3.4	5	4.8	88%

Factor 2	Palatal Length	8	32	45.6	52.4	6.8	3.6	4.6	4.7	70%
Factor 2	Palatal Length	9	32	46.6	50.6	4.1	2.8	3.8	4.2	105%
Factor 2	Palatal Length	10	32	47.1	53	5.9	3.5	4.9	4.7	80%
Factor 2	Palatal Length	11	32	48.2	53.3	5.2	3.1	4.4	5	98%
Factor 2	Palatal Length	12	32	48.3	53.4	5.1	2.7	4.2	4.2	82%
Factor 2	Palatal Length	13	32	49.3	56.1	6.8	4.3	4.1	4.9	72%
Factor 2	Palatal Length	14	32	50.2	58.1	7.9	3.4	7.5	6.5	83%
Factor 2	Palatal Length	15	32	50.5	55.6	5.1	2.4	4	4.6	91%
Factor 2	Palatal Length	16	32	49.9	54.9	5.1	3.1	4.4	5	99%
Factor 2	Palatal Length	17	32	49.1	56.5	7.4	4.1	4.9	5.2	71%
Factor 2	Palatal Length	18	32	49.8	57.2	7.4	3.7	5.6	6.6	88%
Factor 2	Palatal Length	19	32	51.2	56.3	5.1	4.3	2.3	4.2	83%
Factor 2	Palatal Length	20	32	49.9	55.2	5.3	3.7	3.7	4.4	83%
Factor 2	SNA	5	32	77.8	85.5	7.7	4.9	4.7	5.2	68%
Factor 2	SNA	6	32	80.8	84.6	3.9	3.2	2	3.1	81%
Factor 2	SNA	7	32	80.6	84.4	3.8	3.5	3	3.9	102%
Factor 2	SNA	8	32	80	85.5	5.4	3.5	2.5	3.9	72%
Factor 2	SNA	9	32	80.1	84.8	4.8	2.4	3.5	3.2	67%
Factor 2	SNA	10	32	79.7	85.7	6	4.9	4	5.3	88%
Factor 2	SNA	11	32	81.4	85.4	4.1	3	3.5	3.4	83%
Factor 2	SNA	12	32	81.2	86	4.8	2.7	3.8	4.3	89%
Factor 2	SNA	13	32	80.4	86.8	6.5	3.3	3.5	4.1	63%
Factor 2	SNA	14	32	81.1	85.9	4.8	2.9	3.8	4.3	90%
Factor 2	SNA	15	32	80.8	86.1	5.3	3.9	2.1	3.9	73%
Factor 2	SNA	16	32	80.9	85.9	5.1	2.4	4.4	4.5	87%
Factor 2	SNA	17	32	80.4	85.3	4.9	3.2	4.2	4.5	91%
Factor 2	SNA	18	32	81.4	85.8	4.5	3.2	2.6	3.2	72%
Factor 2	SNA	19	32	79.8	85.8	6	3.8	4.3	4.9	81%
Factor 2	SNA	20	32	81.7	85.8	4.1	2.8	3.1	3.4	83%
Factor 3	Gonial Angle	5	32	85.3	92.6	7.3	6.1	10.2	10.3	141%
Factor 3	Gonial Angle	6	32	88.2	99.1	10.9	4.9	6.6	6.9	63%
Factor 3	Gonial Angle	7	32	90.8	102	11.2	5.7	7.1	7.4	66%
Factor 3	Gonial Angle	8	32	91.7	105.1	13.4	6.1	9.5	10	74%
Factor 3	Gonial Angle	9	32	95	103.8	8.8	5.3	8.6	9.5	108%
Factor 3	Gonial Angle	10	32	96.4	109.5	13.1	4.5	11.4	10.7	82%
Factor 3	Gonial Angle	11	32	98.9	108.8	9.9	5.2	10.6	10.6	107%
Factor 3	Gonial Angle	12	32	99.6	109	9.4	5.1	8.2	7.4	78%
Factor 3	Gonial Angle	13	32	102.4	116	13.6	8.2	9.8	11	81%
Factor 3	Gonial Angle	14	32	106.4	118.5	12.1	5.2	11.3	11	91%
Factor 3	Gonial Angle	15	32	104.2	117.8	13.6	6.3	16	15.9	117%
Factor 3	Gonial Angle	16	32	102.7	115.9	13.2	6	7.7	8.2	63%

Factor 3	Gonial Angle	17	32	106.8	117.4	10.6	8.9	11	11.8	112%
Factor 3	Gonial Angle	18	32	104.9	119.8	14.9	7.8	11.1	11.6	78%
Factor 3	Gonial Angle	19	32	109.3	119.5	10.2	9.3	3.9	8.8	87%
Factor 3	Gonial Angle	20	32	107.3	119.1	11.8	5.8	9.4	9.6	81%
Factor 3	Copus length Go-Gn)	5	32	58.9	67.6	8.7	5.8	7.7	8.7	100%
Factor 3	Copus length Go-Gn)	6	32	63.5	71.3	7.8	4	6.5	6.2	79%
Factor 3	Copus length Go-Gn)	7	32	65.1	74.4	9.3	4	6.5	7	75%
Factor 3	Copus length Go-Gn)	8	32	66.2	75.8	9.6	5.8	6.6	6.5	67%
Factor 3	Copus length Go-Gn)	9	32	67.9	74.3	6.3	3.8	6.1	6	95%
Factor 3	Copus length Go-Gn)	10	32	68.4	77.9	9.5	5.9	7.6	8.4	89%
Factor 3	Copus length Go-Gn)	11	32	70.8	78.4	7.6	4.8	9.5	9.5	125%
Factor 3	Copus length Go-Gn)	12	32	71.8	78.3	6.4	4	6.7	6	93%
Factor 3	Copus length Go-Gn)	13	32	73.7	84.1	10.5	5.1	7	7.2	69%
Factor 3	Copus length Go-Gn)	14	32	75.9	85.3	9.4	5.5	9.6	8.9	95%
Factor 3	Copus length Go-Gn)	15	32	74.8	84.4	9.6	6.6	8.6	9.3	96%
Factor 3	Copus length Go-Gn)	16	32	73.4	80.3	6.9	3.3	8.5	8.4	122%
Factor 3	Copus length Go-Gn)	17	32	77	85.2	8.2	5.6	9.3	8.6	105%
Factor 3	Copus length Go-Gn)	18	32	76.1	88.3	12.2	8.1	12.1	13.7	113%
Factor 3	Copus length Go-Gn)	19	32	75.3	85.5	10.2	7.9	6.2	7.2	70%
Factor 3	Copus length Go-Gn)	20	32	76	84.7	8.7	5.8	7.9	9.4	108%
Factor 3	Ramus height	5	32	33.8	38.3	4.5	1.7	3.4	3.4	76%
Factor 3	Ramus height	6	32	35.9	41.3	5.4	3.8	4.4	4.7	87%
Factor 3	Ramus height	7	32	35.8	42.3	6.5	4	4.8	5	77%
Factor 3	Ramus height	8	32	36.9	43.7	6.8	3	5	5.2	77%
Factor 3	Ramus height	9	32	37.8	43.3	5.6	3	4.3	4.4	79%
Factor 3	Ramus height	10	32	38.3	46.5	8.2	5.6	4.6	5.7	70%
Factor 3	Ramus height	11	32	40.3	47.2	6.9	2.8	5.8	5.7	83%
Factor 3	Ramus height	12	32	41.2	47.3	6.1	4.4	4.4	5.2	85%
Factor 3	Ramus height	13	32	42.4	50	7.6	4.4	5.5	6.2	82%
Factor 3	Ramus height	14	32	44.8	51.3	6.6	3.8	5.7	6.1	93%
Factor 3	Ramus height	15	32	41.4	51.4	10	4.1	6.4	7	70%
Factor 3	Ramus height	16	32	45.1	52.3	7.2	5.1	6.4	7	97%
Factor 3	Ramus height	17	32	46.5	54.7	8.2	6.1	6.2	8.1	98%
Factor 3	Ramus height	18	32	44.6	52.8	8.2	4	4.8	5.6	69%
Factor 3	Ramus height	19	32	46.1	52.9	6.8	5.8	5.8	6.7	97%
Factor 3	Ramus height	20	32	44.8	55.7	10.9	6.8	5	7.9	72%
Factor 3	Gonial Angle	5	32	123.2	132.4	9.2	3.9	6.8	6.7	73%
Factor 3	Gonial Angle	6	32	121.3	128.7	7.4	5.2	5.1	5.6	75%
Factor 3	Gonial Angle	7	32	121.4	128.5	7.1	5.9	5.7	6	85%
Factor 3	Gonial Angle	8	32	120.2	127.8	7.6	5.4	6.7	7.4	98%
Factor 3	Gonial Angle	9	32	120.6	129.4	8.8	5.9	5.5	6.9	78%

Factor 3	Gonial Angle	10	32	119.4	128.3	8.9	4.7	5.5	6.1	69%
Factor 3	Gonial Angle	11	32	117.4	127.9	10.5	6.2	5.7	6.2	59%
Factor 3	Gonial Angle	12	32	118.9	126.6	7.8	7.6	5	7.4	95%
Factor 3	Gonial Angle	13	32	118	126.2	8.2	5.2	5.5	6	74%
Factor 3	Gonial Angle	14	32	117.6	126.2	8.6	7.4	4.8	6.9	81%
Factor 3	Gonial Angle	15	32	116.7	124.9	8.2	4.8	4.5	5.9	71%
Factor 3	Gonial Angle	16	32	116	125.1	9.1	4.9	5.7	5.9	65%
Factor 3	Gonial Angle	17	32	114.6	121.1	6.5	4.4	5.4	6.5	101%
Factor 3	Gonial Angle	18	32	116.3	124.3	7.9	6.8	7.6	9	114%
Factor 3	Gonial Angle	19	32	116.1	129.4	13.3	6	8	8.6	65%
Factor 3	Gonial Angle	20	32	114.6	123.4	8.8	6.4	4.5	6.6	75%
Factor 3	SNB	5	32	74.1	79.2	5.1	4.9	2	4.9	96%
Factor 3	SNB	6	32	75.6	79.5	3.8	4.4	3	4.3	112%
Factor 3	SNB	7	32	74.9	80.3	5.4	3.3	3.1	4	74%
Factor 3	SNB	8	32	74.9	80	5.1	4	2.9	4.5	89%
Factor 3	SNB	9	32	75.6	80.3	4.7	2.8	4.5	4.3	91%
Factor 3	SNB	10	32	74.7	81.1	6.4	3.8	2.9	4.3	67%
Factor 3	SNB	11	32	76.4	81.2	4.8	3	3	3.7	75%
Factor 3	SNB	12	32	76.2	81.3	5.2	1.7	3	3.2	62%
Factor 3	SNB	13	32	76.6	81.9	5.3	4.2	2.4	4.3	80%
Factor 3	SNB	14	32	77.4	81.8	4.4	4	4.1	5.4	123%
Factor 3	SNB	15	32	77.4	82.1	4.7	1.9	3.7	3.7	79%
Factor 3	SNB	16	32	76.3	82.3	6	3	4.7	4.4	73%
Factor 3	SNB	17	32	77.7	81.8	4.1	4.1	3.3	5.1	123%
Factor 3	SNB	18	32	77.5	82.6	5.1	3.5	4	4.7	93%
Factor 3	SNB	19	32	74.5	83.9	9.4	6.7	4.9	6.3	67%
Factor 3	SNB	20	32	77.6	82.5	4.9	3.6	4.3	4.5	91%
Factor 4	Palatal plane to Occlusal plane	5	32	13.5	19.8	6.3	3	3.9	5.2	83%
Factor 4	Palatal plane to Occlusal plane	6	32	13.5	18.3	4.8	2.4	2.7	3	61%
Factor 4	Palatal plane to Occlusal plane	7	32	10.6	15.6	5	3.7	2.3	4.1	82%
Factor 4	Palatal plane to Occlusal plane	8	32	9.7	14.7	4.9	3.6	3.9	4.4	89%
Factor 4	Palatal plane to Occlusal plane	9	32	9.4	14.6	5.2	3.5	3.7	3.6	69%
Factor 4	Palatal plane to Occlusal plane	10	32	9.6	13.8	4.2	2.9	3.6	3.4	82%
Factor 4	Palatal plane to Occlusal plane	11	32	7.7	12.1	4.4	3.5	3.8	4.5	103%
Factor 4	Palatal plane to Occlusal plane	12	32	7.8	12.3	4.6	3.3	2.8	3.6	80%

Factor 4	Palatal plane to Occlusal plane	13	32	7.1	12.4	5.3	3.9	3	3.7	70%
Factor 4	Palatal plane to Occlusal plane	14	32	5.4	11.2	5.8	3.9	4.5	5.2	89%
Factor 4	Palatal plane to Occlusal plane	15	32	5.7	10.9	5.2	2.7	4.9	5.3	103%
Factor 4	Palatal plane to Occlusal plane	16	32	5.5	11.5	5.9	3.5	3.8	3.8	64%
Factor 4	Palatal plane to Occlusal plane	17	32	6.3	11.4	5.1	4.3	3.9	4.9	96%
Factor 4	Palatal plane to Occlusal plane	18	32	7.4	10.9	3.4	2.4	3.5	4.2	122%
Factor 4	Palatal plane to Occlusal plane	19	32	5.6	11.4	5.8	2.9	5.9	6.1	104%
Factor 4	Palatal plane to Occlusal plane	20	32	3.9	11.7	7.8	3.8	6.3	6.3	81%
Factor 4	Mandible plan to Occlusal plane	5	32	9.7	15.5	5.8	4.3	4.5	5.5	95%
Factor 4	Mandible plan to Occlusal plane	6	32	10.6	16.6	6	5.3	5.2	5.6	93%
Factor 4	Mandible plan to Occlusal plane	7	32	12.5	17.9	5.4	2.5	5.3	5.4	100%
Factor 4	Mandible plan to Occlusal plane	8	32	13.6	18	4.4	2.9	4	4	91%
Factor 4	Mandible plan to Occlusal plane	9	32	13.8	18.5	4.7	3.5	3.4	4.5	96%
Factor 4	Mandible plan to Occlusal plane	10	32	13.9	19.5	5.6	3	4	4.1	73%
Factor 4	Mandible plan to Occlusal plane	11	32	13.8	20	6.2	3.7	4.3	4.9	78%
Factor 4	Mandible plan to Occlusal plane	12	32	13.8	19	5.3	3.6	3.7	3.9	75%
Factor 4	Mandible plan to Occlusal plane	13	32	14.6	20.3	5.8	4.1	4.2	4.8	84%
Factor 4	Mandible plan to Occlusal plane	14	32	14.8	21	6.2	3.7	5.1	4.7	76%
Factor 4	Mandible plan to Occlusal plane	15	32	15.5	22.2	6.7	4.5	4.4	6.2	92%
Factor 4	Mandible plan to Occlusal plane	16	32	14.5	21	6.5	4.4	4.3	6.4	98%
Factor 4	Mandible plan to Occlusal plane	17	32	13.2	18	4.8	3.7	4.1	4.9	102%
Factor 4	Mandible plan to Occlusal plane	18	32	14.5	18.7	4.2	3.8	3.4	4.7	111%
Factor 4	Mandible plan to Occlusal plane	19	32	15.7	22.2	6.5	3.7	3.9	4.3	66%

Factor 4	Mandible plan to Occlusal plane	20	32	14.7	20.6	5.9	5.6	2.6	5.4	92%
Factor 4	Occlusal plane to SN	5	32	20.9	27.5	6.6	5.2	2	4.8	73%
Factor 4	Occlusal plane to SN	6	32	21.1	26.7	5.6	5.7	4.9	6.9	123%
Factor 4	Occlusal plane to SN	7	32	18.1	24.6	6.5	5.7	3.6	5.6	86%
Factor 4	Occlusal plane to SN	8	32	16.7	23	6.4	3.4	4.6	4.8	75%
Factor 4	Occlusal plane to SN	9	32	17	23.1	6.1	3.9	3.2	4.3	70%
Factor 4	Occlusal plane to SN	10	32	16	22.8	6.8	4.5	3.2	4.6	68%
Factor 4	Occlusal plane to SN	11	32	14.9	20.8	5.8	4.2	3.6	4.6	79%
Factor 4	Occlusal plane to SN	12	32	16	20.2	4.2	3.6	2.7	4	95%
Factor 4	Occlusal plane to SN	13	32	14.7	20.6	5.9	4.3	3.5	4.6	78%
Factor 4	Occlusal plane to SN	14	32	12.8	19.2	6.4	4.8	4.5	5.3	83%
Factor 4	Occlusal plane to SN	15	32	13.7	19.1	5.4	3.2	2.7	3.8	70%
Factor 4	Occlusal plane to SN	16	32	13.1	19	5.9	3.8	5.6	5.4	91%
Factor 4	Occlusal plane to SN	17	32	15	19.7	4.7	2.2	4.6	4.8	102%
Factor 4	Occlusal plane to SN	18	32	14.3	20.3	6	5	3.1	4.8	80%
Factor 4	Occlusal plane to SN	19	32	12.6	22.5	9.8	3	4.9	4.7	48%
Factor 4	Occlusal plane to SN	20	32	11.6	19.9	8.3	3.5	4.1	4.5	54%
Factor 4	Occlusal plane to FH	5	32	11.4	18.1	6.7	5.2	3.6	5.6	83%
Factor 4	Occlusal plane to FH	6	32	8.3	15.9	7.6	4.5	5.4	6.5	85%
Factor 4	Occlusal plane to FH	7	32	7.3	14.6	7.4	5.3	3.5	5.3	72%
Factor 4	Occlusal plane to FH	8	32	6.6	12.9	6.2	3.9	4	4.5	73%
Factor 4	Occlusal plane to FH	9	32	6.5	12.5	6	3.9	4.5	5.7	95%
Factor 4	Occlusal plane to FH	10	32	6.1	11.3	5.2	3.4	3.5	4.1	78%
Factor 4	Occlusal plane to FH	11	32	4.3	10.6	6.3	4.3	4.4	5.3	84%
Factor 4	Occlusal plane to FH	12	32	5.3	9.6	4.3	3.6	2.9	4	91%
Factor 4	Occlusal plane to FH	13	32	3.9	9.6	5.7	4.2	3.2	4.5	78%
Factor 4	Occlusal plane to FH	14	32	2.6	8.7	6.1	4	3.7	4.4	72%
Factor 4	Occlusal plane to FH	15	32	3.1	8.7	5.6	4	4.1	4.5	80%
Factor 4	Occlusal plane to FH	16	32	2.3	8.6	6.3	3.2	5	5.1	81%
Factor 4	Occlusal plane to FH	17	32	3.5	9.1	5.6	3	3.1	4.5	81%
Factor 4	Occlusal plane to FH	18	32	4.4	7.9	3.5	3.9	3.7	4.8	135%
Factor 4	Occlusal plane to FH	19	32	2	11.1	9.1	2.9	6.1	5.8	64%
Factor 4	Occlusal plane to FH	20	32	2.7	7.1	4.4	4.5	4.9	6.4	146%
Factor 5	SN-GoGn	5	32	32	39.5	7.5	3.3	3.9	4.4	58%
Factor 5	SN-GoGn	6	32	32	36.7	4.7	5.7	4.6	5.7	121%
Factor 5	SN-GoGn	7	32	31	37.6	6.6	4.4	4.1	5.2	80%
Factor 5	SN-GoGn	8	32	29.9	37	7.1	4.4	4.6	5.2	74%
Factor 5	SN-GoGn	9	32	29.5	37.1	7.6	6.2	4.3	6.4	84%
Factor 5	SN-GoGn	10	32	29.9	37.6	7.7	4.7	4.8	5.5	72%
Factor 5	SN-GoGn	11	32	29.3	35.9	6.6	5.4	4.1	5.4	82%
Factor 5	SN-GoGn	12	32	28.6	35.7	7.2	6	4.8	5.8	82%

Factor 5	SN-GoGn	13	32	28.1	36.1	8.1	4.6	5.2	5.5	68%
Factor 5	SN-GoGn	14	32	27.2	35.9	8.8	6	5.5	7.5	85%
Factor 5	SN-GoGn	15	32	28.5	36.8	8.3	7	4.1	6.7	80%
Factor 5	SN-GoGn	16	32	27.1	34.8	7.7	5.6	5.2	5.6	73%
Factor 5	SN-GoGn	17	32	25.3	34.7	9.4	4.3	6.2	6.5	69%
Factor 5	SN-GoGn	18	32	27.6	36.5	8.9	6.5	4	6.5	73%
Factor 5	SN-GoGn	19	32	27	40.6	13.6	7.1	6.4	8.7	64%
Factor 5	SN-GoGn	20	32	26	37.4	11.4	9.1	5.4	9.7	85%
Factor 5	Upper Gonial Angle	5	32	50.1	54.7	4.6	1.7	4.5	4.6	101%
Factor 5	Upper Gonial Angle	6	32	46.2	52.6	6.4	2.5	4	4	63%
Factor 5	Upper Gonial Angle	7	32	47.1	53	6	4.3	3.9	5.1	85%
Factor 5	Upper Gonial Angle	8	32	45.8	52.7	6.9	4.2	4.5	4.9	71%
Factor 5	Upper Gonial Angle	9	32	46	52	6	3.5	3.5	3.9	65%
Factor 5	Upper Gonial Angle	10	32	45.6	50.2	4.6	3.6	4.2	4.6	99%
Factor 5	Upper Gonial Angle	11	32	45.2	50.8	5.6	3.2	4.5	5	90%
Factor 5	Upper Gonial Angle	12	32	45.4	50.6	5.2	4.4	3.4	4.4	84%
Factor 5	Upper Gonial Angle	13	32	43.7	49.7	5.9	3.8	4	4.5	75%
Factor 5	Upper Gonial Angle	14	32	43.8	49.8	5.9	3.3	3.4	3.9	66%
Factor 5	Upper Gonial Angle	15	32	42.8	49.5	6.7	3	5	5.3	80%
Factor 5	Upper Gonial Angle	16	32	43.1	48.2	5.1	3.5	3.7	4	79%
Factor 5	Upper Gonial Angle	17	32	42.3	46.3	4	3.5	3.6	4	100%
Factor 5	Upper Gonial Angle	18	32	42.6	48.6	6.1	4	4.1	4.9	81%
Factor 5	Upper Gonial Angle	19	32	44.4	49.3	4.9	4.9	5.3	5.3	108%
Factor 5	Upper Gonial Angle	20	32	42.5	46	3.5	2.3	5	4.7	135%
Factor 5	Lower Gonial Angle	5	32	73.8	78.1	4.3	3.2	3.6	4.3	100%
Factor 5	Lower Gonial Angle	6	32	73.6	78.7	5.2	2.2	3.9	4	77%
Factor 5	Lower Gonial Angle	7	32	72	78.2	6.2	3.9	4.3	4.7	76%
Factor 5	Lower Gonial Angle	8	32	72.7	76.9	4.2	4.6	4.2	5	120%
Factor 5	Lower Gonial Angle	9	32	71.8	78.6	6.8	4.5	4.4	4.7	70%
Factor 5	Lower Gonial Angle	10	32	72.4	78.8	6.4	5.1	3.5	5.5	86%
Factor 5	Lower Gonial Angle	11	32	71.6	77.4	5.8	4.3	4.2	4.5	78%
Factor 5	Lower Gonial Angle	12	32	72.4	77.4	5.1	3.7	3.8	4.7	94%
Factor 5	Lower Gonial Angle	13	32	72.4	77.8	5.4	4.4	5.2	6	110%
Factor 5	Lower Gonial Angle	14	32	72.6	78.9	6.4	6.2	4	6.7	104%
Factor 5	Lower Gonial Angle	15	32	71.8	79.8	8	2.9	5.2	4.3	54%
Factor 5	Lower Gonial Angle	16	32	71.9	77.1	5.3	3.9	4.1	4.6	87%
Factor 5	Lower Gonial Angle	17	32	70.4	76.7	6.3	3.8	4.8	5.3	84%
Factor 5	Lower Gonial Angle	18	32	72.2	77.6	5.3	4	5.8	6.3	117%
Factor 5	Lower Gonial Angle	19	32	71.9	80.2	8.4	7.3	5.5	8.1	97%
Factor 5	Lower Gonial Angle	20	32	71.6	79.3	7.7	6.5	4.6	7.9	102%
Factor 5	Anterior Facial Height	5	32	93.6	101.4	7.8	5	7.3	7.3	93%

Factor 5	Anterior Facial Height	6	32	96.6	108.8	12.2	4.5	11.4	11.4	94%
Factor 5	Anterior Facial Height	7	32	97.7	110.5	12.8	6.1	8.5	9.1	71%
Factor 5	Anterior Facial Height	8	32	100.8	111.7	11	5.5	9.4	9.3	85%
Factor 5	Anterior Facial Height	9	32	103	110.9	7.9	4.9	9.7	9.7	123%
Factor 5	Anterior Facial Height	10	32	105	116	11	6.4	12.1	12.1	110%
Factor 5	Anterior Facial Height	11	32	104.8	116.3	11.5	7.6	8.4	9.2	80%
Factor 5	Anterior Facial Height	12	32	105.6	117.6	12.1	6.2	8.6	8.5	70%
Factor 5	Anterior Facial Height	13	32	109.9	121.7	11.8	6.3	10.7	10.7	91%
Factor 5	Anterior Facial Height	14	32	112.7	125.2	12.6	7.4	10.5	9.8	78%
Factor 5	Anterior Facial Height	15	32	108.2	122.1	13.9	7	13.2	11.3	82%
Factor 5	Anterior Facial Height	16	32	110.7	122.6	11.9	5	10.8	10	84%
Factor 5	Anterior Facial Height	17	32	114	127.9	13.9	7.8	11.1	12.7	91%
Factor 5	Anterior Facial Height	18	32	111.6	126.2	14.6	7.5	11.4	11.6	80%
Factor 5	Anterior Facial Height	19	32	117.4	127.6	10.3	9.4	9.5	10.5	102%
Factor 5	Anterior Facial Height	20	32	114	126.7	12.7	5.3	12.9	12.3	97%
Factor 5	Posterior Face Height	5	32	58.2	63	4.8	5.6	5.7	6	125%
Factor 5	Posterior Face Height	6	32	60.1	67.8	7.8	4.3	6.3	6.7	86%
Factor 5	Posterior Face Height	7	32	61.5	69.9	8.4	3.7	6.7	6.5	77%
Factor 5	Posterior Face Height	8	32	62.9	73	10.1	5.2	8.6	8.7	86%
Factor 5	Posterior Face Height	9	32	63.5	71.9	8.4	4.4	6	6.5	77%
Factor 5	Posterior Face Height	10	32	65.7	76.2	10.5	5.9	7.6	8	76%
Factor 5	Posterior Face Height	11	32	68.7	75.1	6.4	4.2	5.8	6.1	95%
Factor 5	Posterior Face Height	12	32	68.5	77.4	8.9	5.9	5.5	6.8	76%
Factor 5	Posterior Face Height	13	32	70.8	80.8	10.1	6.5	6.1	7.3	73%
Factor 5	Posterior Face Height	14	32	74.4	83.8	9.4	6.1	8.6	8.5	91%
Factor 5	Posterior Face Height	15	32	70	81.5	11.5	5.1	8.9	9.7	84%
Factor 5	Posterior Face Height	16	32	73.8	81.3	7.5	5.2	6	7.8	103%
Factor 5	Posterior Face Height	17	32	76.5	85.2	8.7	6.8	6.5	8.9	102%
Factor 5	Posterior Face Height	18	32	73.1	85.3	12.2	4.8	8.2	8	66%
Factor 5	Posterior Face Height	19	32	73.5	83.6	10.1	4.1	4.8	5.7	56%
Factor 5	Posterior Face Height	20	32	74.6	86.6	12	5.8	9.3	9.7	81%
Factor6	Wits appraisal	5	32	-6.6	0.3	6.9	4.4	5.6	5.7	83%
Factor6	Wits appraisal	6	32	-5.3	-0.2	5.2	5.6	3	5.2	100%
Factor6	Wits appraisal	7	32	-2.6	0.4	3	2.3	2.7	3.3	109%
Factor6	Wits appraisal	8	32	-3	2	5	3.4	3.3	3.9	79%
Factor6	Wits appraisal	9	32	-2.7	0.6	3.3	2.9	2.7	3.5	108%
Factor6	Wits appraisal	10	32	-2.6	1.4	4	2.9	2.7	3.6	90%
Factor6	Wits appraisal	11	32	-1.8	1.8	3.7	2.8	2.4	3.1	85%
Factor6	Wits appraisal	12	32	-2.5	2.3	4.8	3	3.5	3.9	80%
Factor6	Wits appraisal	13	32	-2.6	2.2	4.8	4	3.1	4.4	92%
Factor6	Wits appraisal	14	32	-2.2	2.7	4.8	3.5	4.4	4.7	97%

Factor6	Wits appraisal	15	32	-3.3	2.1	5.4	3.9	3.1	4.3	79%
Factor6	Wits appraisal	16	32	-3.3	2.2	5.5	5.5	4.1	5.7	103%
Factor6	Wits appraisal	17	32	-4.1	2	6.1	3.6	3.1	4.4	72%
Factor6	Wits appraisal	18	32	-5	1.6	6.6	3.8	3.4	4.4	66%
Factor6	Wits appraisal	19	32	-4.4	2.9	7.3	3.9	5.2	5.6	77%
Factor6	Wits appraisal	20	32	-3.4	2.8	6.2	3.6	2.6	3.8	61%
Factor6	Convexity A-NPo	5	32	1.7	6.4	4.7	2.6	4.1	5.1	108%
Factor6	Convexity A-NPo	6	32	2.6	6.8	4.2	3	2.8	3.2	75%
Factor6	Convexity A-NPo	7	32	3	6.3	3.3	2.5	3	3.4	101%
Factor6	Convexity A-NPo	8	32	3.1	6.3	3.2	2.4	3.1	2.9	92%
Factor6	Convexity A-NPo	9	32	3	6	3	2.3	2.6	2.5	83%
Factor6	Convexity A-NPo	10	32	2.4	6.2	3.8	2.3	3.4	3.7	98%
Factor6	Convexity A-NPo	11	32	2.7	5.9	3.2	3.2	2.1	3	93%
Factor6	Convexity A-NPo	12	32	1.9	6.2	4.3	4.1	2.4	3.9	90%
Factor6	Convexity A-NPo	13	32	2.1	6.3	4.3	3.3	3.2	3.5	83%
Factor6	Convexity A-NPo	14	32	1.7	6.1	4.4	2.9	2.9	3.3	74%
Factor6	Convexity A-NPo	15	32	1.3	6.5	5.2	2.2	3.6	3.5	67%
Factor6	Convexity A-NPo	16	32	1.4	5.6	4.2	3.2	3.4	3.7	88%
Factor6	Convexity A-NPo	17	32	1.1	5.3	4.2	2.2	2.9	3.4	82%
Factor6	Convexity A-NPo	18	32	0.9	6.8	5.8	2.4	4	4.3	74%
Factor6	Convexity A-NPo	19	32	0.5	7.3	6.9	3.5	5.8	6.1	88%
Factor6	Convexity A-NPo	20	32	1.9	5.7	3.8	2.5	3.1	4	106%
Factor6	ANB	5	32	2.2	7.4	5.2	2.7	5.8	5	95%
Factor6	ANB	6	32	2.9	7.4	4.6	1.4	3.2	3	66%
Factor6	ANB	7	32	3.5	6.8	3.3	2.4	2.7	3.3	98%
Factor6	ANB	8	32	3.4	7	3.6	2	3	2.8	79%
Factor6	ANB	9	32	3.3	6.2	2.9	2.1	2	2.4	83%
Factor6	ANB	10	32	2.9	6.5	3.6	2.8	2.1	3.1	87%
Factor6	ANB	11	32	3.1	6.2	3.2	2.7	1.8	3	94%
Factor6	ANB	12	32	2.4	6.2	3.8	3.7	2.4	3.6	94%
Factor6	ANB	13	32	2.6	6.5	3.9	2.7	2.5	3.3	83%
Factor6	ANB	14	32	2.2	6.2	4	2.1	2.7	3.2	79%
Factor6	ANB	15	32	2.5	6.1	3.6	3	3.4	3.5	99%
Factor6	ANB	16	32	1.8	5.8	3.9	3.6	2.4	3.9	99%
Factor6	ANB	17	32	1.9	5.1	3.2	1.9	2.4	2.8	88%
Factor6	ANB	18	32	1.4	6.3	4.9	2.8	3.5	3.6	73%
Factor6	ANB	19	32	0.9	6.9	6	3.8	4.3	4.3	71%
Factor6	ANB	20	32	2.3	6.1	3.8	3.3	4.1	5	132%
Factor6	Convexity-NA-Apo	5	32	4.5	15.8	11.3	5.5	10.4	11.7	104%
Factor6	Convexity-NA-Apo	6	32	6	15.9	9.9	6.3	6.5	7.6	76%
Factor6	Convexity-NA-Apo	7	32	6.7	15.4	8.7	6	5.9	7.6	87%

Factor6	Convexity-NA-Apo	8	32	6.9	14.7	7.8	5.4	5.5	6.1	79%
Factor6	Convexity-NA-Apo	9	32	6.8	13.4	6.6	4.4	4.6	5.1	77%
Factor6	Convexity-NA-Apo	10	32	4.7	13.6	8.9	5.3	6.6	7.1	79%
Factor6	Convexity-NA-Apo	11	32	5.9	13.1	7.2	6.6	3.7	6.6	92%
Factor6	Convexity-NA-Apo	12	32	4.3	13.1	8.8	8.7	4.6	8.4	96%
Factor6	Convexity-NA-Apo	13	32	4.4	13.2	8.8	6.7	5.5	7.3	83%
Factor6	Convexity-NA-Apo	14	32	3.4	11.8	8.4	6.1	5.8	6.9	83%
Factor6	Convexity-NA-Apo	15	32	3.1	13	9.9	4.7	7	7.3	73%
Factor6	Convexity-NA-Apo	16	32	3	11.3	8.3	6.6	6.1	7.4	90%
Factor6	Convexity-NA-Apo	17	32	2.4	10.2	7.8	5.3	6.1	7.1	91%
Factor6	Convexity-NA-Apo	18	32	1.9	13.2	11.3	4.9	9.1	8.9	79%
Factor6	Convexity-NA-Apo	19	32	0.9	13.7	12.8	7.4	9.7	10.2	80%
Factor6	Convexity-NA-Apo	20	32	3.3	12.3	9	5.1	6.7	8.7	96%

25th= Percentile 25th, 75th= Percentile 75th; IQR= Interquartilar range
