# Supplementary material for: LOESS-Based Cephalometric Growth Curves for Clinical Assessment of Craniofacial Development: A Cross-Sectional Study
Source: Dent J (Basel). 2026 May 4;14(5):269. doi: 10.3390/dj14050269 (PMC13205012; doi:10.3390/dj14050269)
Supplement: Supplementary file 1 [file dentistry-14-00269-s001.zip › Supplementary Figure S1.pdf]

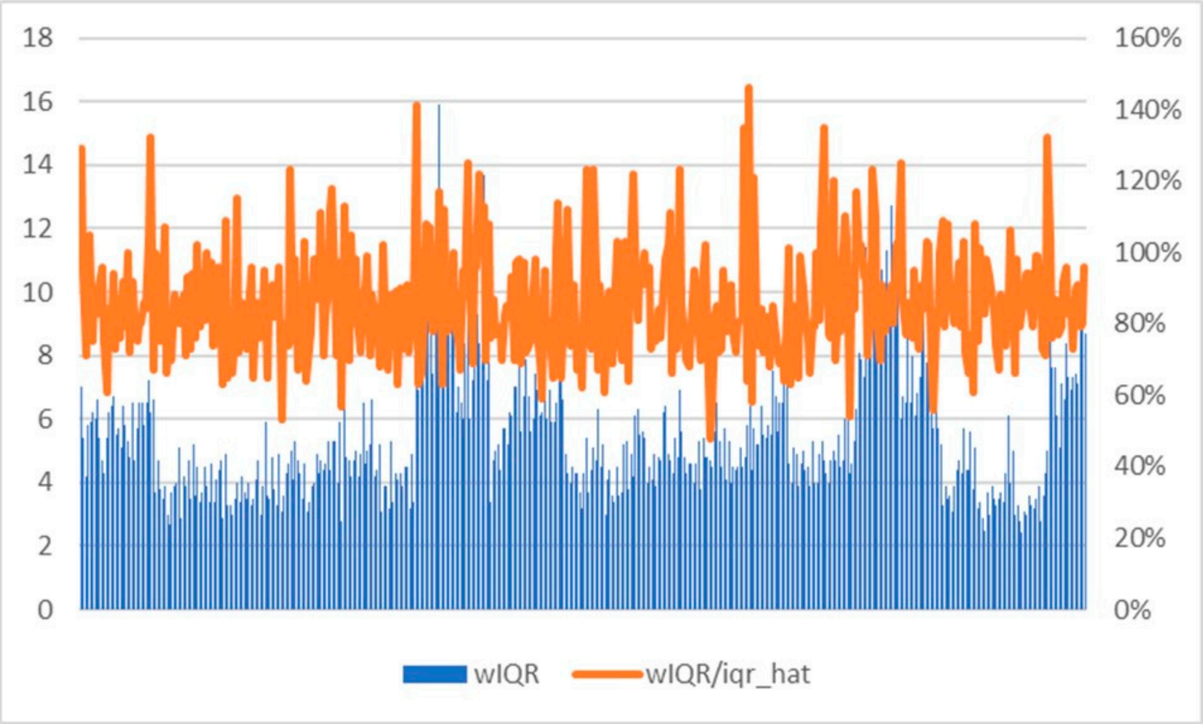

Supplementary Fig. S1. Sample size adequacy assessment using bootstrap-derived interquartile range stability. *This figure summarizes the bootstrap-based stability analysis used to assess the minimum sample size required for each cephalometric variable between 5 and 20 years of age. The blue bars represent the 95% confidence interval width of the interquartile range (wIQR), while the orange line depicts the relative stability index expressed as the ratio between the confidence interval width and the estimated interquartile range (wIQR/IQR), shown as a percentage. Each point corresponds to a specific cephalometric variable at a particular age. This visualization was used to evaluate the precision and stability of the percentile estimates across variables and age groups and to inform sample size adequacy.*
